# Wide resection and stabilization of ulnar stump by extensor carpi ulnaris for giant cell tumor of distal ulna: two case reports

**DOI:** 10.4076/1757-1626-2-8617

**Published:** 2009-07-21

**Authors:** Manjeet Singh, Siddhartha Sharma, Chetan Peshin, Iftikhar H Wani, Agnivesh Tikoo, Sanjeev K Gupta, Dara Singh

**Affiliations:** Department of Orthopedics, Government Medical College(Bakshinagar), Jammu, Jammu & Kashmir - (180001)India

## Abstract

The distal end of ulna is an extremely uncommon site for primary bone tumors in general and giant cell tumor in particular. Wide resection is usually indicated in such cases and at times it may be necessary to remove of a long segment of the distal ulna. Any ulnar resection proximal to the insertion of pronator quadratus can lead to instability in the form of radio-ulnar convergence and dorsal displacement (winging) of the ulnar stump. This can result in diminution of forearm rotation and weakness with grasp. Stabilization of the ulnar stump after resection for a giant cell tumor was described by Kayias & Drosos. We are adding two more cases to the literature. Both patients had excellent functional outcome and there were no instances of recurrence at three years of follow-up.

## Introduction

Giant cell tumour (GCT) of bone is a rare, benign, locally invasive tumour, accounting for approximately 3% to 5% of all primary bone tumours [1]. GCTs of the distal end of the ulna are extremely rare, accounting for approximately 0.45% to 3.2% of all the cases of GCTs [2]. Wide resection of the distal ulna with or without reconstruction or stabilisation of the ulnar stump is the recommended treatment for GCTs in such locations. We present two patients with GCT of the distal ulna, both treated by wide resection of the distal ulna followed by stabilisation of the remaining ulna using one half of the extensor carpi ulnaris (ECU) tendon.

## Case presentation

### Case report 1

A 22-year-old Indian male, painter by occupation, presented to us with a painless swelling along the ulnar aspect of his right distal forearm since the last four months. To begin with, it was the size of a peanut but had gradually increased to its present size. There was no history of any other swelling in the body, fever, loss of weight or appetite, or history of similar complaints in the past. The family, occupational, recreational and drug histories were not significant. The general physical and systemic examinations were within normal limits.

On examination, there was an oval swelling 6 × 4 cm, occupying the distal third of ulna. The overlying skin was of normal colour and temperature. There was no overlying scar, sinus or prominent veins. The swelling was diffusely tender and homogenously firm in consistency. It was free from the overlying skin but adherent to the underlying bone. The range of motion of wrist was normal and painless. The distal neurovascular status was normal and grasping power equal in both hands.

**Figure 1.**
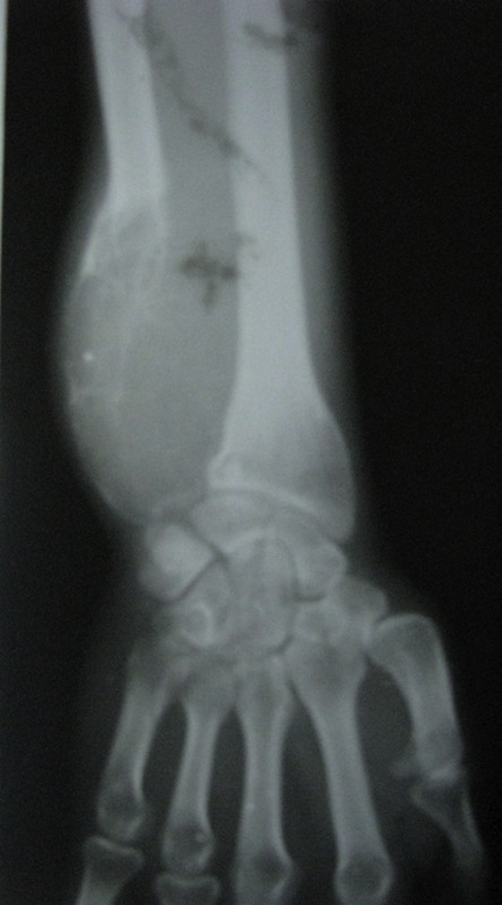
AP Radiograph of right forearm showing an expansile, multiloculated lytic lesion at the distal end of ulna with absence of periosteal reaction.

Serum biochemistry studies were within normal limits. Plain radiographs of the right ulna showed an expansile, multiloculated lytic lesion at its lower end with absence of periosteal reaction (Figure 1). Plain chest radiographs were within normal limits.

Magnetic Resonance Imaging (MRI) study was planned to delineate the extent of soft tissue involvement, but the patient was very poor and chose not to have the investigation.

A clinical diagnosis of GCT was made, which was confirmed post operatively by histopathological examination. The condition, its prognosis and various treatment modalities were discussed at length with the patient.On the basis of clinical and radiographic evaluation, the lesion was graded as Stage 3 (aggressive) as per the Enneking Staging system for benign bone tumors [3]. As per the standard recommendations for Stage 3 lesions, a wide resection was planned after obtaining informed and written consent from the patient. The resection margins were calculated keeping in view the radiological extent of the lesion. Keeping in view the patient’s high functional demands, we also decided to stabilize the ulnar stump using the extensor carpi ulnaris tenodesis technique described by Kayias & Drosos [2].

**Figure 2.**
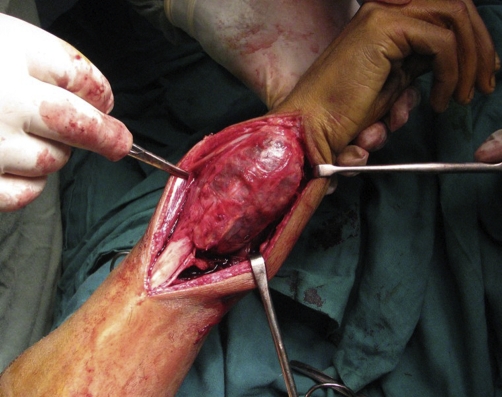
Peroperative photograph showing a large tumor originating from the right ulna.

**Figure 3.**
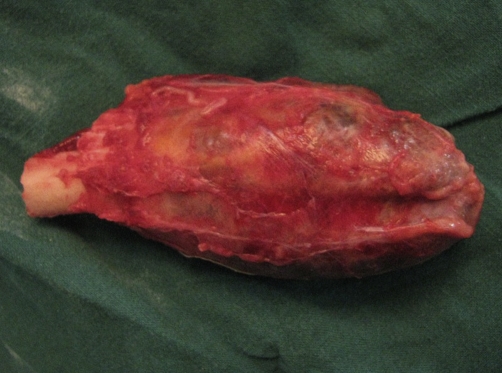
The resected tumor specimen.

**Figure 4.**
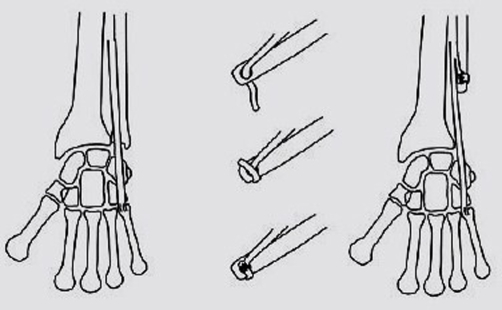
The Extensor Carpi Ulnaris tenodesis technique. Reproduced with permission [14].

The tumor resection was extra-periosteal with 3 cm margin of the normal bone proximal to the tumor. This included approximately half (twelve centimetres) of the distal end of ulna, the triangular fibro cartilage complex, the ulnar border of the pronator quadratus and a part of the distal radio-ulnar joint capsule (Figure 2, 3). The extensor carpi ulnaris (ECU) tendon was dissected free from the tumor mass by blunt dissection and longitudinally split to a point 1 cm proximal to the cut end of the ulna. The tendon was passed through a 3.2 mm drill hole, 5 mm above the end of the ulnar stump in a dorsal to volar direction with the forearm held in supination. The tendon was then directed to the ulnar side and sutured back on itself (Figure 4). This manoeuvre resulted in a cuff of the ECU tendon, which effectively stabilized the ulnar stump. The remnant of distal radio ulnar joint capsule was sutured with the ECU tendon in order to prevent ulnar subluxations of the carpus. The wound was closed in layers over a drain. Postoperatively, the forearm was immobilised in supination using an above elbow splint for two weeks, following which physiotherapy was commenced. Thereafter, the forearm splint was used only at night for another eight weeks and the patient was gradually advised full range of motion at the wrist and elbow.

Follow-up was carried once every three months for the first two years and six monthly thereafter. Functional evaluation was done using the criteria described by Ferracini [4]. The patient had a normal function and muscle strength, no pain and no ulnar instability. He had restriction of forearm pronation - supination of approximately 15 degrees. However, the patient did not seem to be bothered by this and could carry out his professional activity as a painter quite efficiently. This patient therefore scored 16 out of a total of 18 points on the Ferracini scale which is consistent with an excellent functional outcome.

### Case report 2

A 28-year-old Indian male, shopkeeper by occupation, presented to us with history of pain and swelling on the ulnar aspect of the distal part of left forearm since one year. The swelling was painless and had increased in size gradually. There was no history of fever, loss of weight or appetite, swelling at other sites or history of similar complaints in the past. The family, occupational, recreational and drug histories were not significant. The general physical and systemic examinations were within normal limits.

On examination this swelling was comparatively smaller i.e. 3 cm long and 2 cm wide. The overlying skin was normal and free from the swelling. It was firm in consistency and adherent to the ulna. The distal neurovascular status was normal.

**Figure 5.**
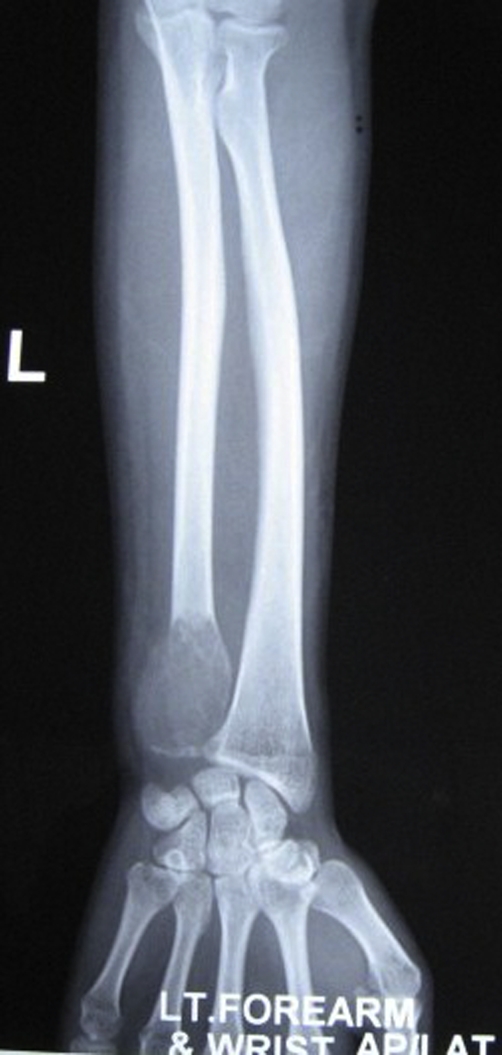
Case #2. AP Radiograph of left forearm showing a lytic lesion of the distal aspect of ulna with an indistinct medial margin and absence of periosteal reaction.

Plain radiographs revealed a lytic lesion of the distal aspect of ulna with an indistinct medial margin (Figure 5). A provisional diagnosis of Enneking stage 3 GCT ulna was made. After discussing the condition, prognosis and treatment options with the patient, we decided to perform wide resection.

We performed a wide resection of the distal 6 cm of the ulna that included 3 cm of the normal bone. ECU stabilization was done in the same fashion as described in Case #1 and the wound was closed over drain.

The post operative protocol was similar as described for Case #1. This patient had normal function and muscle strength, no pain or ulnar instability and full range forearm flexion - extension and pronation - supination. He scored 18 out of 18 points on the Ferracini scale.

**Figure 6.**
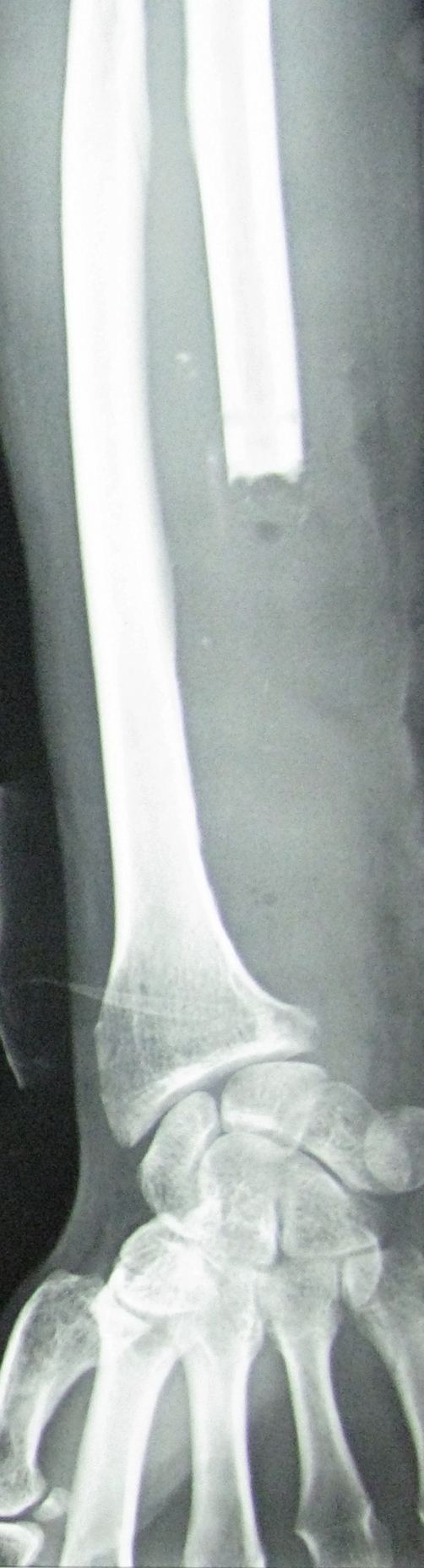
AP radiograph of Case #1 at 3 years of follow up.

**Figure 7.**
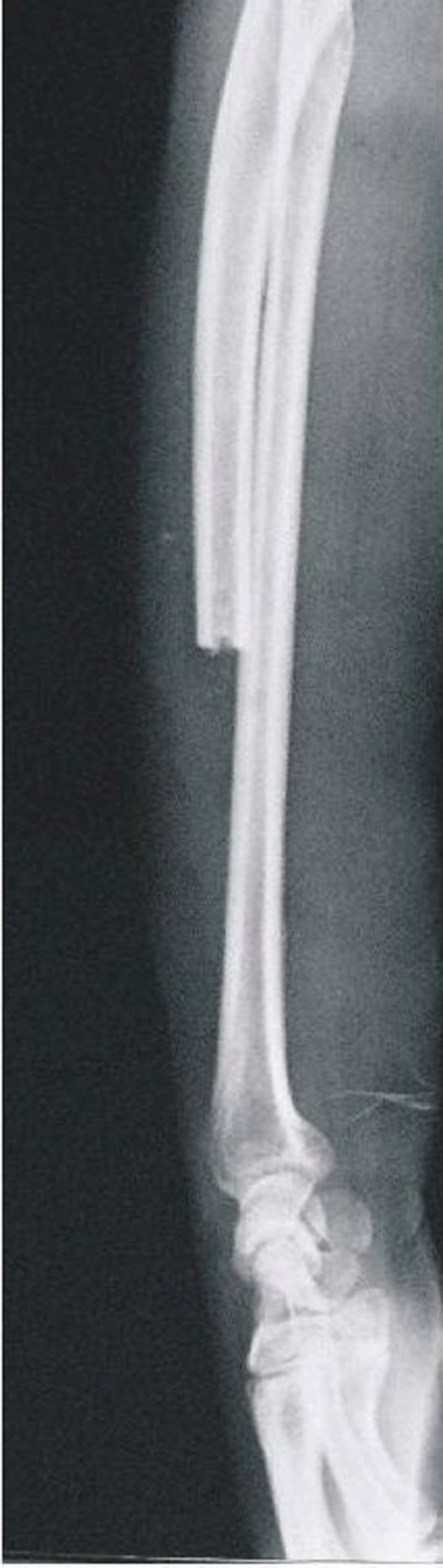
Lateral radiograph of Case #1 at 3 years of follow up.

Histo-pathological examination of the resection specimen in both cases was consistent with the diagnosis of giant cell tumor. We achieved tumor free margins in both cases. Radiographic evaluation was done in by measuring the axis between the long axis of the radius and the third metacarpal on lateral view and ulnar subluxation of the carpus on AP view. Both cases had a normal radiographic outcome (Figure 6, 7). There were no instances of recurrence in either case at three years of follow-up.

## Discussion

The distal end of ulna is a rare site for primary bone tumors, especially giant cell tumour. Tumours at this location can be benign, locally aggressive or malignant. The Enneking staging system for musculoskeletal tumours is useful not only for planning the modality of treatment but also for prognostication. Stage 1 lesions require no treatment, Stage 2 lesions can be managed by extended curettage whereas Stage 3 lesions will need a wide excision [3]. As per this system, most GCTs present as Stage 2 or 3 lesions.

The treatment of giant cell tumors should focus on minimizing the recurrences since it a locally aggressive tumor with a high potential for recurrence. Intra-lesional curettage without adjuvant therapy is associated with a high recurrence rate [5]. It seems that recurrence rates correlate better with the inadequacy of tumor tissue removal rather than the type of specific adjuvant treatment used [6].

The distal ulna has been traditionally considered as dispensable and resection of the distal ulna for degenerative conditions was first described by Darrach and subsequently modified by Dingman [7]. Functionally, the distal end of the ulna helps in forearm rotations (supination and pronation), grip strength and in maintaining the relationship between the carpus and the distal end of the radius. The ulnar collateral ligament of the wrist, which emanates from the ulnar styloid process and the triangular fibrocartilage complex (TFCC), plays an important role in the maintaining of this anatomical relationship [8].

Most of the studies on GCT ulna have focussed on wide resection of the distal ulna. In a study on eight patients with various neoplasms of the distal ulna managed by en bloc resection alone, Cooney et al achieved excellent results in 75% of the cases and concluded that reconstruction of the osseous defect is not routinely indicated [9].

On the other hand, many authors have documented failures after wide excision of the distal ulna. This may be attributable to the fact that the ulnar stump has a tendency to displace in a dorsal direction (winging) and also tends to converge towards the radius. This instability can therefore be a cause of persistent pain, weakness with hand grip and limitation of forearm rotation [10]. Furthermore, removal of the TFCC along with distal ulna may result in ulnar deviation of the carpus due to loss of support by the ulnar collateral ligament support which attaches to the TFCC.

To overcome this problem, many authors have attempted reconstruction or stabilization of the ulnar stump. Gainor et al reported excellent results in two patients treated by a ‘lasso’ tendon graft stabilization of the ulnar stump [11]. In a series of nine cases, Ferracini et al performed a soft tissue stabilization procedure in seven cases using the Flexor Carpi Ulnaris (FCU) stabilization, fascia lata or an autograft. All seven cases had an excellent functional outcome whereas two cases without stabilization had a fair outcome [4]. Hashizume described the ulnar buttress arthroplasty procedure using the autogenous iliac crest bone graft and reported good oncological and functional outcome [12]. Wurapa et al described a two staged reconstructive procedure using allograft and reported good functional outcome at 40 months of follow up. Stabilization using the Extensor Carpi Ulnaris tendon after ulnar resection was originally described by Goldner & Hayes [13]. However, the application of this technique for Giant Cell Tumor of the ulna was first described by Kayias et al. The patient had an excellent oncological and functional outcome [14]. This technique is particularly useful in those cases where there is a possibility of the tumor having invaded the flexor compartment rendering the FCU unsuitable for use.

More recently, Roidis et al have described distal ulnar implant arthroplasty as a definitive treatment for a recurrent GCT of the distal ulna. The patient had a good functional and oncological outcome at two years of follow-up [15].

## Conclusions

Giant cell tumor of the distal ulna is an extremely rare entity; therefore there are no clear cut guidelines about the preferred modality of treatment. However, most of the authors would agree that a good outcome depends upon two factors: wide resection of the tumor in order to minimize recurrences and stabilization of the ulnar stump in order to achieve a good functional outcome. There is not enough evidence to recommend one ulnar stump stabilization procedure over the other. The authors also feel that the treatment should be individualised, taking into consideration the patient’s age, the length of the ulnar resection and patient’s expectation about postoperative function.

## Abbreviations

AP: antero posterior
ECU: extensor carpi ulnaris
FCU: flexor carpi ulnaris
GCT: giant cell tumor
TFCC: triangular fibro cartilaginous complex
